# Group decision-making algorithm with sine trigonometric r,s,t-spherical fuzzy aggregation operators and their application

**DOI:** 10.1038/s41598-024-61382-y

**Published:** 2024-05-11

**Authors:** Muhammad Azeem, Ayesha Ilyas, Jawad Ali, Madiha Ghamkhar, Muhammad I. Syam

**Affiliations:** 1 Department of Mathematics and Statistics, University of Agriculture, Faisalabad, 38000 Pakistan; 2 Institute of Numerical Sciences, Kohat University of Science and Technology, Kohat, KPK Pakistan; 3 Department of Mathematical Sciences, United Arab Emirates University, P. O. Box 15551 Al-Ain, UAE

**Keywords:** r-s-t-spherical fuzzy set, Trigonometric operations, Aggregation operators, MAGDM, Engineering, Mathematics and computing

## Abstract

*r*, *s*, *t*-spherical fuzzy (*r*, *s*, *t*-SPF) sets provide a robust framework for managing uncertainties in decision-making, surpassing other fuzzy sets in their ability to accommodate diverse uncertainties through the incorporation of flexible parameters *r*, *s*, and *t*. Considering these characteristics, this article explores sine trigonometric laws to enhance the applicability and theoretical foundation for *r*, *s*, *t*-SPF setting. Following these laws, several aggregation operators (AOs) are designed for aggregation of the *r*, *s*, *t*-SPF data. Meanwhile, the desired characteristics and relationships of these operators are studied under sine trigonometric functions. Furthermore, we build a group decision-making algorithm for addressing multiple attribute group decision-making (MAGDM) problems using the developed AOs. To exemplify the applicability of the proposed algorithm, we address a practical example regarding laptop selection. Finally, parameter analysis and a comprehensive comparison with existing operators are conducted to uncover the superiority and validity of the presented AOs.

## Introduction

In real-world scenarios, as a system grows in complexity, it becomes increasingly difficult for decision-makers (DnMs) to select the best option among various alternatives. To tackle such scenarios, Zadeh^1^ pioneered the concept of fuzzy sets (FS), designed to handle ambiguous and uncertain human opinions. A fuzzy set assigns a truth membership value (TMV) to each element of the universal set within the range of [0, 1], capturing the degree of unpredictability in the information. In a crisp set, an element either belongs to the universal set or does not, making it a less versatile tool for broadly describing human perspectives. FS theory offers a more versatile system compared to crisp sets because it encompasses all values within the range of [0, 1]. Additionally, Atanassov^2^ introduced the concept of intuitionistic fuzzy sets (IFS), which expanded on FS by incorporating both the TMV and the false membership value (FMV) within the range of [0, 1]. This framework provides a comprehensive way to express human viewpoints positively and negatively. In the IFS model, only specific information in the form of pairs ($0\le m\le 1$ and $0\le n\le 1$) is allowed, ensuring that $0\le m+n\le 1$ for flexibility in selecting TMV and FMV. However, the sum of TMV and FMV can sometimes exceed the [0, 1] range. These limitations restrict the use of IFS in certain decision-making (DM) scenarios when $0\le m+n\le 1$ exceeds [0, 1]. To address such situations, Yager^3^ developed the concept of Pythagorean fuzzy sets (PyFS), which allows $0\le m^2+n^2\le 1$, expanding on its applications. However, there are instances where the sum of the squares of the TMV and FMV exceeds the range of [0, 1], posing a challenge for PyFS to explain such scenarios. To address these cases, Yager^4^ originated the idea of q-rung orthopair fuzzy sets (q-ROFS), which imposes the constraint $0\le m^q+n^q\le 1$. This framework provides a comprehensive approach to handling inconsistencies. It’s worth noting that IFS and PyFS are specific instances of q-ROFS. As the parameter *q* increases in q-ROFS, the range describing the assessment information defined by TMV and FMV expands. Till now, several researchers have actively explored the application of q-ROFS in multi-attribute group DM (MAGDM) problems^5–8^, which highlights the potential of q-ROFS to handle the inherent vagueness associated with DM.

Despite the effectiveness of q-ROFS in addressing numerous complex problems, certain scenarios persist where they struggle to provide adequate solutions. This limitation becomes apparent in voting scenarios, where human perspectives often encompass responses such as “yes,” “no,” “don’t know,” and “refuse.” The aforementioned fuzzy models encounter difficulties in accurately representing these diverse reactions. To address these challenges, Cuong^9^ proposed the innovative concept of “picture fuzzy set” (PFS) as a triplet in the form (m,o,n), where the elements of the triplet denote the TMV, abstinence membership value (AMV), and FMV with the constraint $0\le m+o+n\le 1$. This distinctive framework aims to surpass the constraints of the previous fuzzy models by incorporating visual representations, thus offering a more comprehensive and precise depiction of the human perspective in DM processes. The hypothesis of PFS proves ineffective in numerous experimental scenarios. It’s observed that the condition $0\le m+o+n\le 1$ within PFS prevents the independent allocation of each value *m*, *o*, and *n*. To address this limitation, Mahmood et al.^10^ introduced the notion of spherical FS (SFS), where the restriction $0\le m^2+o^2+n^2\le 1$ significantly broadens the range of TMV, AMV, and FMV lying within [0, 1]. However, the utilization of SFS theory may encounter certain challenges. Mahmood et al.^11^ further extended the application of SFS to DM and medical diagnosis problems, yet it remains less effective for certain triplets. This led to the development of t-spherical fuzzy sets (t-SPFS), as proposed by Ullah et al.^12^, which serves as a generalization of SFS and offers a broader spectrum of solutions for real-world challenges^13–17^. However, in the context of t-SPFS, the term level is employed equitably. In practical applications, TMV, AMV, and FMV may exhibit varying degrees of importance, necessitating the use of different expressions. For example, considering the triplet (0.8, 0.7, 0.6), if we aggregate the 4th power of TMV with the 3rd power of AMV and FMV, the result $(0.8^4+0.7^3+0.6^3 = 0.9686<1)$. Consequently, TMV holds an importance coefficient of 4, while AMV and FMV each hold an importance coefficient of 3 for this dataset. Thus, TMV, AMV, and FMV do not share equal levels of importance. Recognizing the significance of these diverse levels, Ali and Naeem^18^ recently introduced the concept of r,s, t-spherical fuzzy set (r,s, t-SPFS). They elaborated on the foundational concepts of this proposed fuzzy tool and conducted a detailed comparison with existing fuzzy models to underscore the need for its development. Following this, Ali comprehensively explained the parameters involved in his study^19^ and presented a series of Aczel-Alsina aggregation operators along with their associated outcomes. The introduction of the concept of r,s, t-SPFS represents a significant advancement, enabling extensive work within this generalized fuzzy framework.

Aggregation operators (AOs) are mathematical functions or rules used in DM and data analysis to combine multiple individual values or scores into a single aggregated value. These operators play a significant role in aggregating information from different criteria or attributes to derive a comprehensive measure for decision support. Numerous authors have pioneered the concept of AOs in the literature and applied them extensively in MAGDM problems^20–24^. For example, Riaz et al.^25^ put forth a robust q-ring orthopair fuzzy prioritizing AOs with applications in MAGDM. Einstein-prioritized AOs were explored for the evaluation of organization Achievement by Jana and Pal^26^, while Wang et al.^27^ proposed PyF interactive Hamacher power AOs for evaluating Swift service standards using entropy weight. Garg^28^ developed sine trigonometric operational laws (STOLs) and their associated Pythagorean fuzzy AOs. Qiyas and Abdullah^29^ proposed sine trigonometric (ST)-spherical fuzzy (SF) AOs for decision advisory system, and Garg^30^ presented a distinctive q-ROF AO built on trigonometric operations. Furthermore, Qiyas et al.^31^ utilized ST-SF AOs for MAGDM. Several AOs have also been designed explicitly for the t-spherical fuzzy (SPF) information. Mahmood et al.^10^ introduced the t-spherical fuzzy weighted geometric AOs, and Zeng et al.^32^ introduced new AOs by consolidating association probabilities with t-SPFS. Ullah et al.^33^ applied Hamacher operations-based AOs for t-SPFS to assess robot execution, and Farid et al.^34^ explored MAGDM issues using their suggested t-spherical fuzzy dynamic Einstein AOs. Recently, Ali and Naeem^18^ introduced *r*, *s*, *t*-spherical fuzzy AOs and thoroughly justified their required characteristics.

**Motivations**

Operational laws are vital for theoretical frameworks, providing structure and enabling effective functioning. Understanding and analyzing these laws are crucial for developing reliable theories. Recognizing their significance allows for the creation of more resilient frameworks to address modern challenges. Sine trigonometric functions possess unique properties, enhancing DM and information interpretation. Integrating these functions leads to improved analysis and decision outcomes. This study introduces STOL and associated aggregation operators within MAGDM, aiming to enhance DM efficiency. The integration of sine operational laws and AOs introduces a distinctive approach in MAGDM, leveraging the periodicity and symmetry of the sine function. This offers advantages in handling cyclical criteria evaluation and situations with equal-weight criteria in positive and negative directions. The resulting decision outputs are more precise and insightful, recognizing fundamental criteria characteristics and interrelationships.

**Research gap**

The untapped potential for combining r, s, t-SPFS, and ST aggregation tools represents a relatively overlooked area in FS theory. Despite the extensive exploration of FSs and aggregation methodologies, the incorporation of ST tools into the framework of r, s, t-SPFS has not been thoroughly addressed in the existing literature.

**Research questions**

To address this research gap, the following inquiries have been formulated:

1. Foundational operations: What are the fundamental operations conducted by ST aggregation tools within the r, s, t-SPFS framework, and how do these operations facilitate periodic aggregation during the process?
2. Innovative aggregation operators: How do the newly introduced aggregation operators, namely ST-weighted averaging, ST-weighted geometric, ST-ordered weighted averaging, ST-ordered weighted geometric, ST-hybrid averaging, and ST-hybrid geometric, perform within the context of r, s, t-SPFS? What are the distinctive characteristics and exceptional scenarios associated with each operator, and how do they improve the aggregation of intricate information?
3. Validation and efficacy: What methods can be employed to validate the robustness and effectiveness of the proposed approaches, particularly in the realm of MAGDM? How do these approaches contribute to DM scenarios, and what insights do they offer for decision-makers navigating fuzzy and multi-attribute environments?
4. Real-world application: How do the implemented approaches fare when applied to real-world problems? What practical insights and implications can be gleaned from the experimental case study conducted within the framework of MAGDM?

**Contributions **

The main contributions of the proposed study are listed as follows:

1. To explore innovative operational laws for *r*, *s*, *t*-SPFS, called STOLs, establishing the fundamental principles necessary to achieve periodic aggregation within the aggregation process.
2. To devise various *r*, *s*, *t*-SPF AOs, namely ST-r, s, t-SPF weighted averaging, ST-r, s, t-SPF weighted geometric, ST-r, s, t-SPF ordered weighted averaging, ST-r, s, t-SPF ordered weighted geometric, ST-r, s, t-SPF hybrid averaging, and ST-r, s, t-SPF hybrid geometric, and analyze their inherent relationships and key characteristics.
3. To outline a stepwise technique for addressing MAGDM challenges using the introduced STOLs and AOs.
4. To solve a MAGDM problem in order to elucidate the algorithm’s performance.

The rest of the work is structured as follows: Sect. 2 presents some basic knowledge regarding *r*, *s*, *t*-SPFS which is conducive in further advancements. In Sect. 3, STOLs are detailed for *r*, *s*, *t*-SPFSs. In Sect. 4, we present several AOs based on ST *r*, *s*, *t*-SPFNs with some of their essential characteristics. Sect. 5 outlines a MAGDM algorithm utilizing the proposed AOs under *r*, *s*, *t*-SPF setting. Sect. 6 illustrates a case study to discuss the effectiveness of the developed method, conduct sensitivity analysis and comparative study with existing literature. Lastly, Sect. 7 concludes this study.

## Fundamental concepts

In this portion, we discuss several fundamental concepts associated with r,s,t-SPFSs

### Definition 1

^18^ Let *K* be a given nonempty set. Then, an r,s, t-SPFS ${\mathcal {Q}}$ on *K* is defined as follow:

1 $$\begin{aligned} {\mathcal {Q}}=\left\{ k, \langle \rho (k),\chi (k),\varepsilon (k) \rangle |k \in K\right\} , \end{aligned}$$

where $\rho : K \rightarrow \left[ 0,1\right]$, $\chi : K \rightarrow \left[ 0,1\right]$, $\varepsilon : K\rightarrow \left[ 0,1\right]$, indicate the membership, neutral and non-membership grades, respectively. These functions are such that there exist certain natural numbers *r*, *s*, and *t*, satisfying $0\le \rho ^{r}\left( k\right) +\chi ^{s}\left( k\right) +\varepsilon ^{t}\left( k\right) \le 1$ $\forall$ $k \in K$.

For convenience, $\langle \rho (k), \chi (k), \varepsilon (k)\rangle$ is referred to as *r*,*s*,*t*-spherical fuzzy number (*r*, *s*, *t*-SPFN), denoted as ${\mathfrak {L}}=(\rho , \chi ,\varepsilon )$.

For guidance on selecting the values of *r*, *s*, and *t* within the framework of the *r*, *s*, *t*-SPFNs, readers are encouraged to refer to Ref.^19^.

### Definition 2

^18^ Let ${\mathfrak {L}}=\left( \rho , \chi , \varepsilon \right)$ be a r,s,t-SPFN. The indeterminacy degree is defined as

2 $$\begin{aligned} \pi (k)=\root \ell \of {1-\rho ^{r}(k)-\chi ^{s}(k)-\varepsilon ^{t}(k)}, \end{aligned}$$

where $\ell$ represents the least common multiple (LCM) of *r*,*s*, and *t*.

### Definition 3

^18^ Consider two *r*, *s*, *t*-SPFNs denoted as ${\mathfrak {L}}_{1}=\left( \rho _{1}, \chi _{1},\varepsilon _{1}\right)$ and ${\mathfrak {L}}_{2}=\left( \rho _{2},\chi _{2},\varepsilon _{2}\right)$ and ${\Cap } > 0$, then

1. $${\mathfrak {L}}_{1}\oplus {\mathfrak {L}}_{2}$$=$$\biggl (\root r^{*} \of {\rho ^{r^{*}}_1+\rho ^{r^{*}}_2-\rho ^{r^{*}}_1\rho ^{r^{*}}_2},\chi _{1}\chi _{2},\varepsilon _{1}\varepsilon _{2}\biggr )$$;
2. $${\mathfrak {L}}_{1}\otimes {\mathfrak {L}}_{2}$$=$$\biggl (\rho _{1}\rho _{2},\root s^{*} \of {\chi ^{s^{*}}_1+\chi ^{s^{*}}_2-\chi ^{s^{*}}_1\chi ^{s^{*}}_2},\root t^{*} \of {\varepsilon ^{t^{*}}_1+\varepsilon ^{t^{*}}_2-\varepsilon ^{t^{*}}_1\varepsilon ^{t^{*}}_2}\biggr )$$;
3. $${\mathfrak {L}}^{{\Cap }}_1$$=$$\biggl (\rho ^{{\Cap }}_1,\root s^{*} \of {1-\left( 1-\chi ^{s^{*}}_1\right) ^{{\Cap }}}, \root t^{*} \of {1-\left( 1-\varepsilon ^{t^{*}}_1\right) ^{{\Cap }}}\biggr )$$;
4. $${\Cap }{\mathfrak {L}}_{1}$$=$$\biggl (\root r^{*} \of {1-\left( 1-\rho ^{r^{*}}_1\right) ^{{\Cap }}},\chi ^{{\Cap }}_1,\varepsilon ^{{\Cap }}_1\biggr )$$;
5. $${\mathfrak {L}}^{c}_1$$=$$\left( \varepsilon _1,\chi _1,\rho _1\right)$$.

Here, $$r^{*}=\max \left\{ r_1,r_2\right\}$$, $$s^{*}=\max \left\{ s_1,s_2\right\}$$, and $$t^{*}=\max \left\{ t_1,t_2\right\}$$.

### Definition 4

^18^ Let ${\mathfrak {L}}=\left( \rho , \chi ,\varepsilon \right)$ be any *r*, *s*, *t*-SPFN, then the score function is given as follows:

3 $$\begin{aligned} \mathscr {S}\left( {\mathfrak {L}}\right) =\frac{1}{2}\left( 1+\left( \rho ^{r}-\chi ^s-\varepsilon ^{t}\right) \right) . \end{aligned}$$

### Definition 5

^18^ Let ${\mathfrak {L}}=\left( \rho , \chi ,\varepsilon \right)$ be any *r*, *s*, *t*-SPFN, then the accuracy function defined as follows:

4 $$\begin{aligned} \mathscr {A}({\mathfrak {L}})=\frac{1}{2}\left( 1+\left( \rho ^{r}+\chi ^{s}+\varepsilon ^{t}\right) \right) . \end{aligned}$$

### Definition 6

^18^ For any two *r*, *s*, *t*-SPFNs, ${\mathfrak {L}}_{i}=\langle \rho _{i},\chi _{i},\varepsilon _{i}\rangle \left( i=1,2\right)$, the comparison procedure is outlined as follows:

1. If $$\mathscr {S}\left( {\mathfrak {L}}_{1}\right) < \mathscr {S}\left( {\mathfrak {L}}_{2}\right)$$, then $${\mathfrak {L}}_{1} \prec {\mathfrak {L}}_{2}$$;
2. If $$\mathscr {S}\left( {\mathfrak {L}}_{1}\right) > \mathscr {S}\left( {\mathfrak {L}}_{2}\right)$$, then $${\mathfrak {L}}_{1} \succ {\mathfrak {L}}_{2}$$;
3. If $$\mathscr {S}\left( {\mathfrak {L}}_{1}\right) =\mathscr {S}\left( {\mathfrak {L}}_{2}\right)$$, and
  - If $$\mathscr {A}\left( {\mathfrak {L}}_{1}\right) <\mathscr {A}\left( {\mathfrak {L}}_{2}\right)$$, then $${\mathfrak {L}}_{1} \prec {\mathfrak {L}}_{2}$$;
  - If $$\mathscr {A}\left( {\mathfrak {L}}_{1}\right) >\mathscr {A}\left( {\mathfrak {L}}_{2}\right)$$, then $${\mathfrak {L}}_{1} \succ {\mathfrak {L}}_{2}$$;
  - If $$\mathscr {A}\left( {\mathfrak {L}}_{1}\right) =\mathscr {A}\left( {\mathfrak {L}}_{2}\right)$$, then $${\mathfrak {L}}_{1} \simeq {\mathfrak {L}}_{2}$$.

### Definition 7

^18^ Let ${\mathfrak {L}}_{i}=\langle \rho _{i},\chi _{i},\varepsilon _{i}\rangle$ be the group of *n* *r*, *s*, *t*-SPFNs with the weight vector $\varphi =\left( \varphi _{1},\varphi _{2},...,\varphi _{n}\right)$ such that $\sum \limits _{i=1}^{n}\varphi _{i}=1$, then r,s,t-SPFWA and r,s,t-SPFWG operators, are defined as:

5 $$\begin{aligned} r,s,t-SPFWA\left( {\mathfrak {L}}_{1},{\mathfrak {L}}_{2},...,{\mathfrak {L}}_{n}\right) = \Bigg (\root r^{*} \of {1-\prod _{i=1}^{n}\biggl (1-\rho _{i}^{r^{*}}\biggr )^{\varphi _{i}}},\prod _{i=1}^{n}\chi _{i}^{\varphi _{i}}, \prod _{i=1}^{n}\varepsilon _{i}^{\varphi _{i}}\Bigg ), \end{aligned}$$

and

6 $$\begin{aligned} r,s,t-SPFWG\left( {\mathfrak {L}}_{1},{\mathfrak {L}}_{2},...,{\mathfrak {L}}_{n}\right) =\Bigg (\prod _{i=1}^{n} \rho _{i}^{\varphi _{i}},\root s^{*} \of {1-\prod _{i=1}^{n}\biggl (1-\chi _{i}^{s^{*}}\biggr )^{\varphi _{i}}}, \root t^{*} \of {1-\prod _{i=1}^{n}\biggl (1-\varepsilon _{i}^{t^{*}}\biggr )^{\varphi _{i}}}\Bigg ). \end{aligned}$$

## Sine trigonometric operational laws based on r,s,t-SPFSs

This section outlines new operations for *r*, *s*, *t*-SPFSs and examines their fundamental properties.

### Definition 8

Let *K* be a given nonempty set and ${\mathcal {Q}}=\left\{ k, \langle \rho (k),\chi (k),\varepsilon (k) \rangle |k \in K\right\}$ be r,s,t-SPFS. Then the definition of sine trigonometric operational laws of r,s,t-SPFS is followed as:

7 $$\begin{aligned} \sin {\mathcal {Q}}=\Biggl \{\Biggl (k,\sin \biggl (\frac{\pi }{2}\rho \biggr ),\root s^{*} \of {1-\sin ^{s^{*}} \biggl (\frac{\pi }{2}\root s^{*} \of {1-\chi ^{s^{*}}}\biggr )},\root t^{*} \of {1-\sin ^{t^{*}} \biggl (\frac{\pi }{2}\root t^{*} \of {1-\varepsilon ^{t^{*}}}\biggr )}\Biggr ):k \in K\Biggr \}, \end{aligned}$$

where $\sin \biggl (\frac{\pi }{2}\rho \biggr )$,$\root s^{*} \of {1-\sin ^{s^{*}}\biggl (\frac{\pi }{2}\root s^{*} \of {1-\chi ^{s^{*}}}\biggr )}$ and $\root t^{*} \of {1-\sin ^{t^{*}}\biggl (\frac{\pi }{2}\root t^{*} \of {1-\varepsilon ^{t^{*}}}\biggr )}\Biggr )$, be the TMV, AMV and FMV, respectively.

### Definition 9

Let ${\mathfrak {L}}=\left( \rho ,\chi ,\varepsilon \right)$ be any r,s,t-SPFN. If

8 $$\begin{aligned} \sin {\mathcal {Q}}=\Biggl \{\Biggl (k,\sin \biggl (\frac{\pi }{2}\rho \biggr ),\root s^{*} \of {1-\sin ^{s^{*}} \biggl (\frac{\pi }{2}\root s^{*} \of {1-\chi ^{s^{*}}}\biggr )},\root t^{*} \of {1-\sin ^{t^{*}} \biggl (\frac{\pi }{2}\root t^{*} \of {1-\varepsilon ^{t^{*}}}\biggr )}\Biggr ):k \in K\Biggr \}. \end{aligned}$$

The function $\sin {\mathcal {Q}}$ operates as a sine trigonometric operator, and its resultant value is referred to as the sine trigonometric r,s,t-SPFN (ST-r,s,t-SPFN).

### Theorem 1

Let ${\mathfrak {L}}_{1}=\left( \rho _{1},\chi _{1},\varepsilon _{1}\right)$, ${\mathfrak {L}}_{2}=\left( \rho _{2},\chi _{2},\varepsilon _{2}\right)$, ${\mathfrak {L}}_{3}=\left( \rho _{3},\chi _{3},\varepsilon _{3}\right)$ be three r,s,t-SPFNs.

1. $$\sin {\mathfrak {L}}_{1}\oplus \sin {\mathfrak {L}}_{2}=\sin {\mathfrak {L}}_{2}\oplus \sin {\mathfrak {L}}_{1}$$,
2. $$\sin {\mathfrak {L}}_{1}\otimes \sin {\mathfrak {L}}_{2}=\sin {\mathfrak {L}}_{2}\otimes \sin {\mathfrak {L}}_{1}$$,
3. $$\left( \sin {\mathfrak {L}}_{1}\oplus \sin {\mathfrak {L}}_{2}\right) \oplus \sin {\mathfrak {L}}_{3}=\sin {\mathfrak {L}}_{1} \oplus \left( \sin {\mathfrak {L}}_{2} \oplus \sin {\mathfrak {L}}_{3}\right)$$,
4. $$\left( \sin {\mathfrak {L}}_{1}\otimes \sin {\mathfrak {L}}_{2}\right) \otimes \sin {\mathfrak {L}}_{3}=\sin {\mathfrak {L}}_{1} \otimes \left( \sin {\mathfrak {L}}_{2} \oplus \sin {\mathfrak {L}}_{3}\right)$$.

### Proof

These can be easily verified from Definition 8. $\square$

### Theorem 2

Let ${\mathfrak {L}}_{1}=\left( \rho _{1},\chi _{1},\varepsilon _{1}\right) , {\mathfrak {L}}_{2}=\left( \rho _{2},\chi _{2},\varepsilon _{2}\right)$ be two r,s,t-SPFNs and ${\Cap },{\Cap }_{1},{\Cap }_{2}>0$ be three real numbers. Then

1. $${\Cap }\left( \sin {\mathfrak {L}}_{1} \oplus \sin {\mathfrak {L}}_{2}\right) ={\Cap } \sin {\mathfrak {L}}_{1} \oplus {\Cap } \sin {\mathfrak {L}}_{2}$$
2. $${\Cap } \sin {\mathfrak {L}}_{2}\left( \sin {\mathfrak {L}}_{1} \otimes \sin {\mathfrak {L}}_{2}\right) ^{\Cap }=\left( \sin {\mathfrak {L}}_{1}\right) ^{\Cap }\otimes \left( \sin {\mathfrak {L}}_{2}\right) ^{\Cap }$$
3. $${\Cap }_{1}\sin {\mathfrak {L}}_{1}\oplus {\Cap }_{2}\sin {\mathfrak {L}}_{1}=\left( {\Cap }_{1}+{\Cap }_{2}\right) \sin {\mathfrak {L}}_{1}$$
4. $$\left( \sin {\mathfrak {L}}_{1}\right) ^{{\Cap }_{1}} \otimes \left( \sin {\mathfrak {L}}_{2}\right) ^{{\Cap }_{2}} =\left( \sin {\mathfrak {L}}_{1}\right) ^{{\Cap }_{1}+{\Cap }_{2}}$$
5. $$\biggl (\left( \sin {\mathfrak {L}}_{1}\right) ^{{\Cap }_{1}}\biggr )^{{\Cap }_{2}}=\left( \sin {\mathfrak {L}}_{1}\right) ^{{\Cap }_{1}{\Cap }_{2}}$$

### Proof

In this theorem, we will verify the proof for 1 and 3, as the rest ones can be determined similarly.For two r,s,t-SPFNs $\left( \rho _{i},\chi _{i},\varepsilon _{i}\right)$ and three real numbers ${\Cap },{\Cap }_{1},{\Cap }_{2} >0$.

Take $\mathscr {X}_{i}=\sin ^{r^{*}}\left( \frac{\pi }{2}\rho _{i}\right)$, $\mathscr {Y}_{i}=\sin ^{s^{*}}\biggl (\frac{\pi }{2}\root s^{*} \of {1-\chi ^{s^{*}}_{i}}\biggr )$ and $\mathscr {Z}_{i}=\sin ^{t^{*}}\biggl (\frac{\pi }{2}\root t^{*} \of {1-\varepsilon ^{t^{*}}_{i}}\biggr )$. By using Definition 9, we have $\sin {\mathfrak {L}}_{i}=\left( \root t^{*} \of {\mathscr {X}_{i}},{\root s^{*} \of {1-\mathscr {Y}_{i}},\root t^{*} \of {1-\mathscr {Z}_{i}}}\right)$ for $i=1,2$, and by OLs between two r,s,t-SPFNs, we have $\sin {\mathfrak {L}}_{1} \oplus \sin {\mathfrak {L}}_{2}=\left( \root r^{*} \of {1-\left( 1-\mathscr {X}_{1}\right) \left( 1-\mathscr {X}_{2}\right) }, \root s^{*} \of {1-\mathscr {Y}_{1}}.\root s^{*} \of {1-\mathscr {Y}_{2}}, \root t^{*} \of {1-\mathscr {Z}_{1}}.\root t^{*} \of {1-\mathscr {Z}_{2}}\right)$

1. $\forall {\Cap } \in {\mathbb {R}}$ then ${\Cap }>0$, we have

$${\Cap }\left( \sin {\mathfrak {L}}_{1} \oplus \sin {\mathfrak {L}}_{2}\right)$$

=$$\biggl (\root r^{*} \of {1-\left( 1-\mathscr {X}_{1}\right) ^{{\Cap }}\left( 1-\mathscr {X}_{2}\right) ^{{\Cap }}},\bigg (\Bigl (\root s^{*} \of {1-\mathscr {Y}_{1}}\root s^{*} \of {1-\mathscr {Y}_{2}}\Bigr )^{{\Cap }}\bigg ),\bigg (\Bigl (\root t^{*} \of {1-\mathscr {Z}_{1}}\root t^{*} \of {1-\mathscr {Z}_{2}}\Bigr )^{{\Cap }}\bigg )$$

=$$\biggl (\root r^{*} \of {1-\left( 1-\mathscr {X}_{1}\right) ^{{\Cap }}},\Bigl (\root s^{*} \of {1-\mathscr {Y}_{1}}\Bigr )^{{\Cap }},\Bigl (\root t^{*} \of {1-\mathscr {Z}_{1}}\Bigr )^{{\Cap }}\biggr )$$ $$\oplus \biggl (\root r^{*} \of {1-\left( 1-\mathscr {X}_{2}\right) ^{{\Cap }}},\Bigl (\root s^{*} \of {1-\mathscr {Y}_{2}}\Bigr )^{{\Cap }},\Bigl (\root t^{*} \of {1-\mathscr {Z}_{2}}\Bigr )^{{\Cap }}\biggr )$$

=$${\Cap } \sin {\mathfrak {L}}_{1} \oplus {\Cap } \sin {\mathfrak {L}}_{2}$$.

3. For real ${\Cap }_{1},{\Cap }_{2}>0$, we have

$${\Cap }_{1}\sin {\mathfrak {L}}_{1} \oplus {\Cap }_{2}\sin {\mathfrak {L}}_{1}$$

=$$\biggl (\root r^{*} \of {1-\left( 1-\mathscr {X}_{1}\right) ^{{\Cap }_{1}}},\Bigl (\root s^{*} \of {1-\mathscr {Y}_{1}}\Bigr )^{{\Cap }_{1}}, \Bigl (\root t^{*} \of {1-\mathscr {Z}_{1}}\Bigr )^{{\Cap }_{1}}\biggr )$$ $$\oplus \biggl (\root r^{*} \of {1-\left( 1-\mathscr {X}_{1}\right) ^{{\Cap }_{2}}},\Bigl (\root s^{*} \of {1-\mathscr {Y}_{1}}\Bigr )^{{\Cap }_{2}},\Bigl (\root t^{*} \of {1-\mathscr {Z}_{1}}\Bigr )^{{\Cap }_{2}}\biggr )$$

=$$\biggl (\root r^{*} \of {1-\left( 1-\mathscr {X}_{1}\right) ^{{\Cap }_{1}+{\Cap }_{2}}},\Bigl (\root s^{*} \of {1-\mathscr {Y}_{1}}\Bigr ) ^{{\Cap }_{1}+{\Cap }_{2}},\Bigl (\root t^{*} \of {1-\mathscr {Z}_{1}}\Bigr )^{{\Cap }_{1}+{\Cap }_{2}}\biggr )$$

=$$\left( {\Cap }_{1}+{\Cap }_{2}\right) \sin {\mathfrak {L}}_{1}$$. $$\square$$

### Theorem 3

For any two r,s,t-SPFNs ${\mathfrak {L}}=\left( \rho ,\chi ,\varepsilon \right)$, ${\mathfrak {B}}=\left( \rho _{{\mathfrak {B}}},\chi _{{\mathfrak {B}}},\varepsilon _{{\mathfrak {B}}}\right)$ such that $\rho \ge \rho _{{\mathfrak {B}}}$ , $\chi \le \chi _{{\mathfrak {B}}}$ and $\varepsilon \le \varepsilon _{{\mathfrak {B}}}$, then $\sin {\mathfrak {L}} \ge \sin {\mathfrak {B}}$.

### Proof

For any ${\mathfrak {L}}=\left( \rho ,\chi ,\varepsilon \right)$, ${\mathfrak {B}}=\left( \rho _{{\mathfrak {B}}},\chi _{{\mathfrak {B}}},\varepsilon _{{\mathfrak {B}}}\right)$ in order that $\rho \ge \rho _{{\mathfrak {B}}}$. As “sine” is an increasing function in $\left[ 0,\frac{\pi }{2}\right]$, So $\sin \left( \frac{\pi }{2}\rho \right) \ge \sin \left( \frac{\pi }{2}\rho _{{\mathfrak {B}}}\right)$. Likewise, for $\chi \le \chi _{{\mathfrak {B}}}$ and $\varepsilon \le \varepsilon _{{\mathfrak {B}}}$ which means that $\sin \biggl (\frac{\pi }{2}\root s^{*} \of {1-\chi ^{s^{*}}}\biggr ) \ge \sin \biggl (\frac{\pi }{2}\root s^{*} \of {1-\chi ^{s^{*}}_{{\mathfrak {B}}}}\biggr ), \sin \biggl (\frac{\pi }{2}\root t^{*} \of {1-\varepsilon ^{t^{*}}}\biggr ) \ge \sin \biggl (\frac{\pi }{2}\root t^{*} \of {1-\varepsilon ^{t^{*}}_{{\mathfrak {B}}}}\biggr )$

$$\Rightarrow$$ $$\root s^{*} \of {1-\sin ^{s^{*}}\biggl (\frac{\pi }{2}\root s^{*} \of {1-\chi ^{s^{*}}}\biggr )}$$ $$\le$$ $$\root s^{*} \of {1-\sin ^{s^{*}}\biggl (\frac{\pi }{2}\root s^{*} \of {1-\chi ^{s^{*}}_{{\mathfrak {B}}}}\biggr )}$$

,$$\root t^{*} \of {1-\sin ^{t^{*}}\biggl (\frac{\pi }{2}\root t^{*} \of {1-\varepsilon ^{s^{*}}}\biggr )}$$ $$\le$$ $$\root t^{*} \of {1-\sin ^{t^{*}}\biggl (\frac{\pi }{2}\root t^{*} \of {1-\varepsilon ^{s^{*}}_{{\mathfrak {B}}}}\biggr )}$$.

Definition 3 has evolved; thus, we enhance $\sin {\mathfrak {L}} \ge \sin {\mathfrak {B}}$ $\square$

### Theorem 4

For r,s,t-SPFNs ${\mathfrak {L}}_{i}$=$\left( \rho _{i}.\chi _{i},\varepsilon _{i}\right)$ and ${\mathfrak {L}}=\left( \rho ,\chi ,\varepsilon \right)$ we have $\sin {\mathfrak {L}}_{i} \oplus \sin {\mathfrak {L}} \ge \sin {\mathfrak {L}}_{i} \otimes \sin {\mathfrak {L}}$.

### Proof

For r,s,t-SPFNs ${\mathfrak {L}}_{i}$ and ${\mathfrak {L}}$. Take $\mathscr {X}=\sin ^{r^{*}}\left( \frac{\pi }{2}\rho \right) ,\mathscr {X}_{i}=\sin ^{r^{*}}\left( \frac{\pi }{2}\rho _{i}\right) , \mathscr {Y}=\sin ^{s^{*}}\biggl (\frac{\pi }{2}\root s^{*} \of {1-\chi ^{s^{*}}}\biggr ),\mathscr {Y}_{i}=\sin ^{s^{*}} \biggl (\frac{\pi }{2}\root s^{*} \of {1-\chi ^{s^{*}}_{i}}\biggr ),\mathscr {Z}=\sin ^{t^{*}}\biggl (\frac{\pi }{2}\root t^{*} \of {1-\varepsilon ^{t^{*}}}\biggr )$ and $\mathscr {Z}_{i}=\sin ^{t^{*}}\biggl (\frac{\pi }{2}\root t^{*} \of {1-\varepsilon ^{t^{*}}_{i}}\biggr )$, we have

9 $$\begin{aligned} \sin {\mathfrak {L}}_{i} \oplus \sin {\mathfrak {L}}=\left( \root r^{*} \of {1-\left( 1-\mathscr {X}_{i}\right) \left( 1-\mathscr {X}\right) }, \root s^{*} \of {1-\mathscr {Y}_{i}}.\root s^{*} \of {1-\mathscr {Y}}, \root t^{*} \of {1-\mathscr {Z}_{i}}.\root t^{*} \of {1-\mathscr {Z}}\right) , \end{aligned}$$

and

10 $$\begin{aligned} \sin {\mathfrak {L}}_{i} \otimes \sin {\mathfrak {L}} =\left( \root r^{*} \of {\mathscr {X}_{i}.\mathscr {X}},\root s^{*} \of {1-\mathscr {Y}_{i}.\mathscr {Y}},\root t^{*} \of {\mathscr {Z}_{i}\mathscr {Z}}\right) . \end{aligned}$$

Since $\mathscr {X},\mathscr {X}_{i},\mathscr {Y},\mathscr {Y}_{i},\mathscr {Z},\mathscr {Z}_{i} \in \left[ 0,1\right]$, we have $\frac{\mathscr {Y}+\mathscr {Y}_{i}}{2} \ge \mathscr {Y}.\mathscr {Y}_{i}$

$\Rightarrow$ $1-\left( 1-\mathscr {Y}\right) \left( 1-\mathscr {Y}_{i}\right) \ge \mathscr {Y}.\mathscr {Y}_{i}$ and thus $\root s^{*} \of {1-\left( 1-\mathscr {Y}\right) \left( 1-\mathscr {Y}_{i}\right) } \ge \root s^{*} \of {\mathscr {Y}.\mathscr {Y}_{i}}$ Alike $\root t^{*} \of {1-\mathscr {Z}_{i}\mathscr {Z}} \ge \root t^{*} \of {1-\mathscr {Z}_{i}}. \root t^{*} \of {1-\mathscr {Z}}$.

Definition 3 and Eqs. (9) and (10) leads to the intended outcome. $\square$

### Theorem 5

For any r,s,t-SPFN ${\mathfrak {L}}$ and real number ${\Cap } >0$, ${\Cap } \sin {\mathfrak {L}} \ge \left( \sin {\mathfrak {L}}\right) ^{{\Cap }}$ iff ${\Cap } \ge 1$ and ${\Cap } \sin {\mathfrak {L}} \le \left( \sin {\mathfrak {L}}\right) ^{{\Cap }}$ iff $0<{\Cap } \le 1$.

### Proof

Based on the previous results, one can easily get the proof. $\square$

## Aggregation operators based on Sine trigonometric r,s,t-SPFNs

We construct the subsequent weighted and geometric AOs relying on STOLs of r,s,t-SPFNs.

Let $\sigma$ be the collection of r,s,t-SPFNs ${\mathfrak {L}}_{i}=\left( \rho _{i},\chi _{i},\varepsilon _{i}\right)$. After that, we label it $\mathscr {X}_{i}=\sin ^{r^{*}}\left( \frac{\pi }{2}\rho _{i}\right)$, $\mathscr {Y}_{i}=\sin ^{s^{*}}\biggl (\frac{\pi }{2}\root s^{*} \of {1-\chi ^{s^{*}}_{i}}\biggr )$ and $\mathscr {Z}_{i}=\sin ^{t^{*}}\biggl (\frac{\pi }{2}\root t^{*} \of {1-\varepsilon ^{t^{*}}_{i}}\biggr )$

### Definition 10

Let ${\mathfrak {L}}_{i}=\left( \rho _{i},\chi _{i},\varepsilon _{i}\right)$ be the group of “n” r,s,t-SPFNs, for $(i=1,2,...,n)$. A ST-r,s,t-SPFWA is a mapping: $\sigma ^{n} \rightarrow \sigma$ and defined as;

11 $$\begin{aligned} ST-r,s,t-SPFWA\left( {\mathfrak {L}}_{1},{\mathfrak {L}}_{2},...,{\mathfrak {L}}_{n}\right) =\varphi _{1}\sin {\mathfrak {L}}_{1}\oplus \varphi _{2}\sin {\mathfrak {L}}_{2}\oplus ...\oplus \varphi _{n}\sin {\mathfrak {L}}_{n}, \end{aligned}$$

where $\varphi =\left( \varphi _{1},\varphi _{2},...,\varphi _{n}\right) ^{T}$ is the weight vector of $\sin {\mathfrak {L}}_{i}$ with $\varphi _{1}>0$ and $\sum \limits _{i=1}^{n}\varphi _{i}=1$.

### Theorem 6

Let ${\mathfrak {L}}_{i}=\left( \rho _{i},\chi _{i},\varepsilon _{i}\right)$ be the group of “n” r,s,t-SPFNs. Then, the aggregated value obtained by ST-r,s,t-SPFWA operator is also r,s,t-SPFN, and this is represented by

12 $$\begin{aligned} ST-r,s,t-SPFWA\left( {\mathfrak {L}}_{1},{\mathfrak {L}}_{2},...,{\mathfrak {L}}_{n}\right) =\Biggl (\root r^{*} \of {1- \prod _{i=1}^{n}\left( 1-\mathscr {X}_{i}\right) ^{w_{i}}},\prod _{i=1}^{n}\left( \root s^{*} \of {1-\mathscr {Y}_{i}}\right) ^{w_{i}},\prod _{i=1}^{n}\left( \root t^{*} \of {1-\mathscr {Z}_{i}}\right) ^{w_{i}}\Biggr ). \end{aligned}$$

### Proof

By applying r,s,t-SPFNs operating rules and subsequently the meaning of $\sin {\mathfrak {L}}$, we can obtain the result in Eq. (12). $\square$

${\textbf {Property 1.}}$ If altogether r,s,t-SPFNs ${\mathfrak {L}}_{i}$=${\mathfrak {L}}$, then ST-r,s,t-SPFWA$\left( {\mathfrak {L}}_{1},{\mathfrak {L}}_{2},...,{\mathfrak {L}}_{n}\right) =\sin {\mathfrak {L}}$.

### Proof

Since ${\mathfrak {L}}_{i}$=${\mathfrak {L}}$ $\forall$ i and therefore $\varphi _{i}{\mathfrak {L}}_{i}=\varphi _{i}{\mathfrak {L}}$ So, by $\sum \limits _{i=1}^{n}\varphi _{i}=1$ and Eq. (12), we have ST-r,s,t-SPFWA$\left( {\mathfrak {L}}_{1},{\mathfrak {L}}_{2},...,{\mathfrak {L}}_{n}\right)$=$\sum \limits _{i=1}^{n}\varphi _{i}\sin {\mathfrak {L}}=\sin {\mathfrak {L}}$. $\square$

${\textbf {Property 2.}}$ If ${\mathfrak {L}}_{i}=\left( \rho _{i},\chi _{i},\varepsilon _{i}\right) ,{\mathfrak {L}}^{-}=\left( \min _{i}\left\{ \rho _{i}\right\} ,\max _{i} \left\{ \chi _{i}\right\} ,\max _{i}\left\{ \varepsilon _{i}\right\} \right)$ and ${\mathfrak {L}}^{+}=\left( \max _{i}\left\{ \rho _{i}\right\} ,\min _{i} \left\{ \chi _{i}\right\} ,\min _{i}\left\{ \varepsilon _{i}\right\} \right)$ be n r,s,t-SPFNs, then $\sin {\mathfrak {L}}^{-} \le$ST-r,s,t-SPFWA$\left( {\mathfrak {L}}_{1},{\mathfrak {L}}_{2},...,{\mathfrak {L}}_{n}\right) \le \sin {\mathfrak {L}}^{+}$.

### Proof

$\forall$ *i*, $\min _{i}\left\{ \rho _{i}\right\} \le \rho _{i} \le max_{i}\left\{ \rho _{i}\right\} , \min _{i}\left\{ \chi _{i}\right\} \le \chi _{i} \le \max _{i}\left\{ \chi _{i}\right\}$ and $\min _{i}\left\{ \varepsilon _{i}\right\} \le \varepsilon _{i} \le \max _{i}\left\{ \varepsilon _{i}\right\}$. This indicates that ${\mathfrak {L}}^{-} \le {\mathfrak {L}}_{i} \le {\mathfrak {L}}^{+}$. Let ST-r,s,t-SPFWA$\left( {\mathfrak {L}}_{1},{\mathfrak {L}}_{2},...,{\mathfrak {L}}_{n}\right) =\sin {\mathfrak {L}}$=$\left( \rho _{{\mathfrak {L}}}, \chi _{{\mathfrak {L}}},\varepsilon _{{\mathfrak {L}}}\right)$. $\sin {\mathfrak {L}}^{-}=\left( \rho _{{{\mathfrak {L}}}^{-}},\chi _{{{\mathfrak {L}}}^{+}},\varepsilon _{{{\mathfrak {L}}}^{+}}\right)$, $\sin {\mathfrak {L}}^{+}=\left( \rho _{{{\mathfrak {L}}}^{+}},\chi _{{{\mathfrak {L}}}^{-}},\varepsilon _{{{\mathfrak {L}}}^{-}}\right)$. Then, relying on the precision of STF , we have $\mathscr {X}_{{{\mathfrak {L}}_{i}}^{-}}=\sin \left( \frac{\pi }{2}\min _{i}\left\{ \rho _{i}\right\} \right) \le \sin \left( \frac{\pi }{2}\rho _{i}\right) =\mathscr {X}_{{{\mathfrak {L}}}_{i}}$ and $\mathscr {X}_{{{\mathfrak {L}}_{i}}^{+}}=\sin \left( \frac{\pi }{2}\max _{i}\left\{ \rho _{i}\right\} \right) \ge \sin \left( \frac{\pi }{2}\rho _{i}\right) =\mathscr {X}_{i}$. Hence,

$$\rho _{{\mathfrak {L}}}$$=$$\root s^{*} \of {1-\prod \limits _{i=1}^{n}\left( 1-\mathscr {X}_{{{\mathfrak {L}}}_{i}}\right) ^{\varphi _{i}}} \ge \root s^{*} \of {1-\prod \limits _{i=1}^{n}\left( 1-\mathscr {X}_{{{{\mathfrak {L}}}_{i}}^{-}}\right) ^{\varphi _{i}}}$$=$$\rho _{{{\mathfrak {L}}}^{-}}$$ and

$$\rho _{{\mathfrak {L}}}$$=$$\root s^{*} \of {1-\prod \limits _{i=1}^{n}\left( 1-\mathscr {X}_{{{\mathfrak {L}}}_{i}}\right) ^{\varphi _{i}}} \le \root s^{*} \of {1-\prod \limits _{i=1}^{n}\left( 1-\mathscr {X}_{{{{\mathfrak {L}}}_{i}}^{+}}\right) ^{\varphi _{i}}}$$=$$\rho _{{{\mathfrak {L}}}^{+}}$$.

Thus, $\rho _{{{\mathfrak {L}}}^{-}} \le \rho _{{\mathfrak {L}}} \le \rho _{{{\mathfrak {L}}}^{+}}$. Also we can get, $\chi _{{{\mathfrak {L}}}^{-}} \le \chi _{{\mathfrak {L}}} \le \chi _{{{\mathfrak {L}}}^{+}}$ and $\varepsilon _{{{\mathfrak {L}}}^{-}} \le \varepsilon _{{\mathfrak {L}}} \le \varepsilon _{{{\mathfrak {L}}}^{+}}$. Thus, we get the proof following Definition 3. $\square$

${\textbf {Property 3.}}$ For r,s,t-SPFNs ${\mathfrak {L}}_{i}$=$\left( \rho _{i},\chi _{i},\varepsilon _{i}\right)$ and ${\mathfrak {L}}_{i}^{'}$=$\left( \rho _{i}^{'},\chi _{i}^{'},\varepsilon _{i}^{'}\right)$. If $\rho _{i} \le \rho _{i}^{'},\chi _{i} \ge \chi _{i}^{'},\varepsilon _{i} \ge \varepsilon _{i}^{'}$ then, $ST-r,s,t-SPFWA\left( {\mathfrak {L}}_{1},{\mathfrak {L}}_{2},...,{\mathfrak {L}}_{n}\right)$ $\le$ $ST-r,s,t-SPFWA\left( {\mathfrak {L}}_{1}^{'},{\mathfrak {L}}_{2}^{'},...,{\mathfrak {L}}_{n}^{'}\right)$.

### Proof

Based on previous results, it can be easily verified. $\square$

### Definition 11

Consider a group ${\mathfrak {L}}_{i}=\left( \rho _{i},\chi _{i},\varepsilon _{i}\right)$ comprising “n” r, s, t-SPFNSs and let ST-r,s,t-SPFWG:$\sigma ^{n} \rightarrow \sigma$, if

13 $$\begin{aligned} \begin{aligned} ST-r,s,t-SPFWG\left( {\mathfrak {L}}_{1},{\mathfrak {L}}_{2},...,{\mathfrak {L}}_{n}\right) = \left( \sin {\mathfrak {L}}_{1}\right) ^{\varphi _{1}} \otimes \left( \sin {\mathfrak {L}}_{2}\right) ^{\varphi _{2}} \otimes ...\otimes \left( \sin {\mathfrak {L}}_{n}\right) ^{\varphi _{n}}\\ =\Biggl (\root r^{*} \of {\prod _{i=1}^{n}\left( \mathscr {X}_{i}\right) ^{\varphi _{i}}},\root s^{*} \of {1-\prod _{i=1}^{n}\left( \mathscr {Y}_{i}\right) ^{\varphi _{i}}},\root t^{*} \of {1-\prod _{i=1}^{n}\left( \mathscr {Z}_{i}\right) ^{\varphi _{i}}}\Biggr ). \end{aligned} \end{aligned}$$

This function is called a sine trigonometric r,s,t-SPFWG operator.

### Definition 12

Consider a group ${\mathfrak {L}}_{i}=\left( \rho _{i},\chi _{i},\varepsilon _{i}\right)$ comprising “n” r, s, t-SPFNSs. A sine trigonometric r,s,t-spherical fuzzy ordered weighted average (ST-r,s,t-SPFOWA) operator is a mapping ST-r,s,t-SPFOWA: $\sigma ^{n} \rightarrow \sigma$, defined as

14 $$\begin{aligned} \begin{aligned} ST-r,s,t-SPFOWA\left( {\mathfrak {L}}_{1},{\mathfrak {L}}_{2},...,{\mathfrak {L}}_{n}\right) = \varphi _{1}\left( \sin {\mathfrak {L}}_{{\theta }{(1)}}\right) \oplus \varphi _{2}\left( \sin {\mathfrak {L}}_{{\theta }{(2)}}\right) \oplus ...\oplus \varphi _{n}\left( \sin {\mathfrak {L}}_{{\theta }{(n)}}\right) \\ =\Biggl (\root r^{*} \of {1-\prod _{i=1}^{n}\left( 1-\mathscr {X}_{{\theta }{(i)}}\right) ^{\varphi _{i}}},\root s^{*} \of {\prod _{i=1}^{n} \left( 1-\mathscr {Y}_{{\theta }{(i)}}\right) ^{\varphi _{i}}},\root t^{*} \of {\prod _{i=1}^{n}\left( 1-\mathscr {Z}_{{\theta }{(i)}}\right) ^{\varphi _{i}}}\Biggr ). \end{aligned} \end{aligned}$$

where $\mathscr {X}_{{\theta }{(i)}}=\sin ^{r^{*}}\left( \frac{\pi }{2}\rho _{{\theta }{(i)}}\right)$ , $\mathscr {Y}_{{\theta }{(i)}}=\sin ^{s^{*}}\left( \frac{\pi }{2}\root s^{*} \of {1-\chi ^{s^{*}}_{{\theta }{(i)}}}\right)$ , $\mathscr {Z}_{{\theta }{(i)}}=\sin ^{t^{*}}\left( \frac{\pi }{2}\root t^{*} \of {1-\varepsilon ^{t^{*}}_{{\theta }{(i)}}}\right)$, $\varphi _{i} >0$ , $\sum \limits _{i=1}^{n}\varphi _{i}=1$ and $\theta$ is the sequence of $\left( 1,2,...,n\right)$ and so that, ${\mathfrak {L}}_{{\theta }{(i-1)}} \ge {\mathfrak {L}}_{{\theta }{(i)}}$ for i=2,3,...,n.

### Definition 13

A sine trigonometric r,s,t-spherical fuzzy ordered weighted geometric (ST-r,s,t-SPFOWG) operator is a mapping ST-r,s,t-SPFOWG: $\sigma ^{n} \rightarrow \sigma$ defined as

15 $$\begin{aligned} \begin{aligned} ST-r,s,t-SPFOWG\left( {\mathfrak {L}}_{1},{\mathfrak {L}}_{2},...,{\mathfrak {L}}_{n}\right) = \left( \sin {\mathfrak {L}}_{{\theta }{(1)}}\right) ^{\varphi _{1}} \otimes \left( \sin {\mathfrak {L}}_{{\theta }{(2)}}\right) ^{\varphi _{2}} \otimes ...\otimes \left( \sin {\mathfrak {L}}_{{\theta }{(n)}}\right) ^{\varphi _{n}} \\ =\Biggl (\Biggl (\prod _{i=1}^{n}\left( \mathscr {X}_{{\theta }{(i)}}\right) ^{\varphi _{i}}\Biggr ),\root s^{*} \of {1-\prod _{i=1}^{n} \left( \mathscr {Y}_{{\theta }{(i)}}\right) ^{\varphi _{i}}},\root t^{*} \of {1-\prod _{i=1}^{n}\left( \mathscr {Z}_{{\theta }{(i)}}\right) ^{\varphi _{i}}}\Biggr ), \end{aligned} \end{aligned}$$

where $\theta$ is the list of permutations.

### Definition 14

A sine trigonometric r,s,t-SPF hybrid average (ST-r,s,t-SPFHA) operator is a mapping ST-r,s,t-SPFHA: $\sigma ^{n} \rightarrow \sigma$, such that $\varphi =\left( \varphi _{1},\varphi _{2},...,\varphi _{n}\right) ^{T}$, with $\varphi _{i} >0$ and $\sum \limits _{i=1}^{n}\varphi _{i}=1$, and

16 $$\begin{aligned} \begin{aligned} ST-r,s,t-SPFHA\left( {\mathfrak {L}}_{1},{\mathfrak {L}}_{2},...,{\mathfrak {L}}_{n}\right) = \varphi _{1}\left( \sin {\mathfrak {L}}_{{\theta }{(1)}}\right) \oplus \varphi _{2}\left( \sin {\mathfrak {L}}_{{\theta }{(2)}}\right) \oplus ...\oplus \varphi _{n}\left( \sin {\mathfrak {L}}_{{\theta }{(n)}}\right) \\ =\Biggl (\root r^{*} \of {1-\prod _{i=1}^{n}\left( 1-\mathscr {X}_{{\theta }{(i)}}\right) ^{\varphi _{i}}}, \root s^{*} \of {\prod _{i=1}^{n}\left( 1-\mathscr {Y}_{{\theta }{(i)}}\right) ^{\varphi _{i}}}, \root t^{*} \of {\prod _{i=1}^{n}\left( 1-\mathscr {Z}_{{\theta }{(i)}}\right) ^{\varphi _{i}}}\Biggr ), \end{aligned} \end{aligned}$$

where ${\mathfrak {L}}_{i}=n\varphi _{i}i$, $\theta$ and $\mathscr {X}_{{\theta }{(i)}}=\sin ^{r^{*}}\left( \frac{\pi }{2}\rho _{{\theta }{(i)}}\right)$ , $\mathscr {Y}_{{\theta }{(i)}}=\sin ^{s^{*}}\left( \frac{\pi }{2}\root s^{*} \of {1-\chi ^{s^{*}}_{{\theta }{(i)}}}\right)$ , $\mathscr {Z}_{{\theta }{(i)}}=\sin ^{t^{*}}\left( \frac{\pi }{2}\root t^{*} \of {1-\varepsilon ^{t^{*}}_{{\theta }{(i)}}}\right)$, is the list of permutations such that, ${\mathfrak {L}}_{{\theta }{(i-1)}} \ge {\mathfrak {L}}_{{\theta }{(i)}}$ for i=2,3,...,n.

### Definition 15

A sine trigonometric r,s,t-SPF hybrid geometric (ST-r,s,t-SPFHG) operator is a mapping ST-r,s,t-SPFHG: $\sigma ^{n} \rightarrow \sigma$, such that $\varphi =\left( \varphi _{1},\varphi _{2},...,\varphi _{n}\right) ^{T}$, with $\varphi >0$ and $\sum \limits _{i=1}^{n}\varphi _{i}=1$, and

17 $$\begin{aligned} \begin{aligned} ST-r,s,t-SPFHG\left( {\mathfrak {L}}_{1},{\mathfrak {L}}_{2},...,{\mathfrak {L}}_{n}\right) = \left( \sin {\mathfrak {L}}_{{\theta }{(1)}}\right) ^{\varphi _{1}} \otimes \left( \sin {\mathfrak {L}}_{{\theta }{(2)}}\right) ^{\varphi _{2}} \otimes ...\otimes \left( \sin {\mathfrak {L}}_{{\theta }{(n)}}\right) ^{\varphi _{n}} \\ =\Biggl (\root r^{*} \of {1-\prod _{i=1}^{n}\left( \mathscr {X}_{{\theta }{(i)}}\right) ^{\varphi _{i}}},\root s^{*} \of {1-\prod _{i=1}^{n} \left( \mathscr {Y}_{{\theta }{(i)}}\right) ^{\varphi _{i}}},\root t^{*} \of {1-\prod _{i=1}^{n}\left( \mathscr {Z}_{{\theta }{(i)}}\right) ^{\varphi _{i}}}\Biggr ), \end{aligned} \end{aligned}$$

here, ${\mathfrak {L}}_{i}$=${\mathfrak {L}}^{n{\varphi _{i}}}$ and $\theta$ is the list of permutations (1,2,3,..,n).

### Basic characteristics of the proposed AOs

This part explores the diverse connections among the proposed AOs along with key elements.

#### Proposition 1

For $x_{i} \ge 0$,$y_{i} >0$ and $z_{i}>0$ with $\sum \limits _{i=1}^{n}y_{i}=1$ and $\sum \limits _{i=1}^{n}z_{i}=1$, we have $\prod \limits _{i=1}^{n}x_{i}y_{i}z_{i} \le \sum \limits _{i=1}^{n}z_{i}y_{i}x_{i}$ if $x_{1}=x_{2}=...=x_{n}$ then the equality is true.

#### Proposition 2

Let $0 \le x,y,z \le 1$ and $0\le p \le 1$, then $0 \le xp+y(1-p)+z(1-p) \le 1$.

#### Theorem 7

From r,s,t-SPFNs ${\mathfrak {L}}_{i}$, the operatives ST-r,s,t-SPFWG satisfy the inequalities and ST-r,s,t-SPFWA$\left( {\mathfrak {L}}_{1},{\mathfrak {L}}_{2},...,{\mathfrak {L}}_{n}\right) \ge$ ST-r,s,t-SPFWG$\left( {\mathfrak {L}}_{1},{\mathfrak {L}}_{2},...,{\mathfrak {L}}_{n}\right)$ where equality holds if ${\mathfrak {L}}_{1}={\mathfrak {L}}_{2}=...={\mathfrak {L}}_{n}$.

#### Proof

For “n” r,s,t-SPFNs ${\mathfrak {L}}_{i}=\left( \rho _{i},\chi _{i},\varepsilon _{i}\right)$ and normalized weight vector $\varphi _{i}>0$, and taking $\mathscr {X}_{i}=\sin ^{r^{*}}\left( \frac{\pi }{2}\rho _{i}\right)$, $\mathscr {Y}_{i}=\sin ^{s^{*}}\biggl (\frac{\pi }{2}\root s^{*} \of {1-\chi ^{s^{*}}_{i}}\biggr )$ and $\mathscr {Z}_{i}=\sin ^{t^{*}}\biggl (\frac{\pi }{2}\root t^{*} \of {1-\varepsilon ^{t^{*}}_{i}}\biggr )$, we have ST-r,s,t-SPFWA$\left( {\mathfrak {L}}_{1},{\mathfrak {L}}_{2},...,{\mathfrak {L}}_{n}\right)$= $\Biggl (\root r^{*} \of {1-\prod \limits _{i=1}^{n}\left( 1-\mathscr {X}_{i}\right) ^{\varphi _{i}}},\root s^{*} \of {\prod \limits _{i=1}^{n} \left( 1-\mathscr {Y}_{i}\right) ^{\varphi _{i}}},\root t^{*} \of {\prod \limits _{i=1}^{n}\left( 1-\mathscr {Z}_{i}\right) ^{\varphi _{i}}}\Biggr )$ and

ST-r,s,t-SPFWG$$\left( {\mathfrak {L}}_{1},{\mathfrak {L}}_{2},...,{\mathfrak {L}}_{n}\right)$$= $$\Biggl (\root r^{*} \of {\prod \limits _{i=1}^{n}\left( \mathscr {X}_{i}\right) ^{\varphi _{i}}},\root s^{*} \of {1-\prod \limits _{i=1}^{n} \left( \mathscr {Y}_{i}\right) ^{\varphi _{i}}},\root t^{*} \of {1-\prod \limits _{i=1}^{n}\left( \mathscr {Z}_{i}\right) ^{\varphi _{i}}}\Biggr )$$

For $\varphi _{i} ,\mathscr {X}_{i},\mathscr {Y}_{i},\mathscr {Z}_{i} \in \left[ 0,1\right]$ and from Proposition 1, we get $1-\prod \limits _{i=1}^{n}\left( 1-\mathscr {X}_{i}\right) ^{\varphi _{i}} \ge 1-\sum \limits _{i=1}^{n}{\varphi _{i}}\left( 1-\mathscr {Y}_{i}\right) \ge 1-\sum \limits _{i=1}^{n}\varphi _{i}\left( 1-\mathscr {Z}_{i}\right) =\sum \limits _{i=1}^{n}\varphi _{i}\mathscr {X}_{i} \ge \prod \limits _{i=1}^{n}\left( \mathscr {X}_{i}\right) ^{\varphi _{i}}$ it means that

18 $$\begin{aligned} \root r^{*} \of {1-\prod _{i=1}^{n}\left( 1-\mathscr {X}_{i}\right) ^{\varphi _{i}}} \ge \root s^{*} \of {\prod _{i=1}^{n}\left( \mathscr {Y}_{i}\right) ^{\varphi _{i}}} \ge \root t^{*} \of {\prod _{i=1}^{n}\left( \mathscr {Z}_{i}\right) ^{\varphi _{i}}} \end{aligned}$$

Likewise, for $\varphi _{i}>0, \mathscr {Y}_{i},\mathscr {Z}_{i} \in \left[ 0,1\right]$ and from Proposition 1, we have the option to obtain

19 $$\begin{aligned} \root t^{*} \of {\prod _{i=1}^{n}\left( 1-\mathscr {Z}_{i}\right) ^{\varphi _{i}}} \le \root r^{*} \of {\prod _{i=1}^{n}\left( \mathscr {X}_{i}\right) ^{\varphi _{i}}} \le \root s^{*} \of {\prod _{i=1}^{n}\left( \mathscr {Y}_{i}\right) ^{\varphi _{i}}} \end{aligned}$$

Hence, using Eqs. (18) and (19), we derive the outcome. $\square$

#### Theorem 8

Let ${\mathfrak {L}}_{i},{\mathfrak {L}}$ are r,s,t-spherical fuzzy numbers, then

1. ST-r,s,t-SPFWA$\left( {\mathfrak {L}}_{1}\oplus {\mathfrak {L}},{\mathfrak {L}}_{2}\oplus {\mathfrak {L}},...,{\mathfrak {L}}_{n}\oplus {\mathfrak {L}}\right)$ $\ge$ ST-r,s,t-SPFWA$\left( {\mathfrak {L}}_{1}\otimes {\mathfrak {L}},{\mathfrak {L}}_{2}\otimes {\mathfrak {L}},...,{\mathfrak {L}}_{n}\otimes {\mathfrak {L}}\right)$.
2. ST-r,s,t-SPFWG$\left( {\mathfrak {L}}_{1}\oplus {\mathfrak {L}},{\mathfrak {L}}_{2}\oplus {\mathfrak {L}},...,{\mathfrak {L}}_{n}\oplus {\mathfrak {L}}\right)$ $\ge$ ST-r,s,t-SPFWG$\left( {\mathfrak {L}}_{1}\otimes {\mathfrak {L}},{\mathfrak {L}}_{2}\otimes {\mathfrak {L}},...,{\mathfrak {L}}_{n}\otimes {\mathfrak {L}}\right)$.

#### Proof

Let ${\mathfrak {L}}_{i},{\mathfrak {L}}$ are r,s,t-SPFNs, then by their operational laws, we get ${\mathfrak {L}}_{i}\oplus {\mathfrak {L}} \ge {\mathfrak {L}}_{i}\otimes {\mathfrak {L}}$ $\forall i$. Utilizing the ST-r,s,t-SPFWA operator’s monotonicity property, we achieve the required result. $\square$

#### Theorem 9

For r,s,t-SPFNs ${\mathfrak {L}}_{i},{\mathfrak {L}}$, we have

1. ST-r,s,t-SPFWA$\left( {\mathfrak {L}}_{1},{\mathfrak {L}}_{2},...,{\mathfrak {L}}_{n}\right) \oplus \sin {\mathfrak {L}} \ge$ ST-r,s,t-SPFWA$\left( {\mathfrak {L}}_{1},{\mathfrak {L}}_{2},...,{\mathfrak {L}}_{n}\right) \otimes \sin {\mathfrak {L}}$.
2. ST-r,s,t-SPFWG$\left( {\mathfrak {L}}_{1},{\mathfrak {L}}_{2},...,{\mathfrak {L}}_{n}\right) \oplus \sin {\mathfrak {L}} \ge$ ST-r,s,t-SPFWG$\left( {\mathfrak {L}}_{1},{\mathfrak {L}}_{2},...,{\mathfrak {L}}_{n}\right) \otimes \sin {\mathfrak {L}}$.

#### Proof

For r,s,t-SPFNs ${\mathfrak {L}}_{i},{\mathfrak {L}}$ the result obtained using the operators of ST-r,s,t-SPFWA, ST-r,s,t-SPFWAG and $\sin {\mathfrak {L}}$ are again r,s,t-SPFNs. Thus, we derived results by applying Theorem 5. $\square$

#### Theorem 10

For r,s,t-SPFNs ${\mathfrak {L}}_{i},{\mathfrak {L}}$, and a real number ${\Cap } \in [0,1]$

1. $${\Cap }$$ST-r,s,t-SPFWA$$\left( {\mathfrak {L}}_{1},{\mathfrak {L}}_{2},...,{\mathfrak {L}}_{n}\right) \oplus \sin {\mathfrak {L}} \ge$$ $$\left( ST-r,s,t-SPFWA\left( {\mathfrak {L}}_{1},{\mathfrak {L}}_{2},...,{\mathfrak {L}}_{n}\right) \right) ^{{\Cap }} \otimes \sin {\mathfrak {L}}$$.
2. $${\Cap }$$ST-r,s,t-SPFWG$$\left( {\mathfrak {L}}_{1},{\mathfrak {L}}_{2},...,{\mathfrak {L}}_{n}\right) \oplus \sin {\mathfrak {L}} \ge$$ $$\left( ST-r,s,t-SPFWG\left( {\mathfrak {L}}_{1},{\mathfrak {L}}_{2},...,{\mathfrak {L}}_{n}\right) \right) ^{{\Cap }} \otimes \sin {\mathfrak {L}}$$.
3. $$\left( ST-r,s,t-SPFWA\left( {\mathfrak {L}}_{1},{\mathfrak {L}}_{2},...,{\mathfrak {L}}_{n}\right) \right) ^{{\Cap }} \oplus \sin {\mathfrak {L}}$$ $$\ge$$ $${\Cap }$$ST-r,s,t-SPFWA$$\left( {\mathfrak {L}}_{1},{\mathfrak {L}}_{2},...,{\mathfrak {L}}_{n}\right) \otimes \sin {\mathfrak {L}}$$.
4. $$\left( ST-r,s,t-SPFWG\left( {\mathfrak {L}}_{1},{\mathfrak {L}}_{2},...,{\mathfrak {L}}_{n}\right) \right) ^{{\Cap }} \oplus \sin {\mathfrak {L}}$$ $$\ge$$ $${\Cap }$$ST-r,s,t-SPFWG$$\left( {\mathfrak {L}}_{1},{\mathfrak {L}}_{2},...,{\mathfrak {L}}_{n}\right) \otimes \sin {\mathfrak {L}}$$.

#### Proof

Let ${\mathfrak {L}}_{i}$, and ${\mathfrak {L}}$ be any two r,s,t-SPFNs and ${\Cap } \in [0,1]$, where ${\Cap }$ be any real number. Subsequently, we possess

${\Cap }$ST-r,s,t-SPFWA$\left( {\mathfrak {L}}_{1},{\mathfrak {L}}_{2},...,{\mathfrak {L}}_{n}\right)$ =$\Biggl (\root r^{*} \of {1-\prod \limits _{i=1}^{n}\left( 1-\mathscr {X}_{i}\right) ^{{\Cap }\varphi _{i}}},\root s^{*} \of {\prod \limits _{i=1}^{n} \left( 1-\mathscr {Y}_{i}\right) ^{{\Cap }\varphi _{i}}},\root t^{*} \of {\prod \limits _{i=1}^{n}\left( 1-\mathscr {Z}_{i}\right) ^{{\Cap }\varphi _{i}}}\Biggr )$ and $\left( ST-r,s,t-SPFWA\left( {\mathfrak {L}}_{1},{\mathfrak {L}}_{2},...,{\mathfrak {L}}_{n}\right) \right) ^{{\Cap }}$

=$$\Biggl (\Biggl (\root r^{*} \of {1-\prod \limits _{i=1}^{n}\left( 1-\mathscr {X}_{i}\right) ^{\varphi _{i}}}\Biggr )^{{\Cap }},\root s^{*} \of {1-\Biggl (1-\prod \limits _{i=1}^{n}\left( 1-\mathscr {Y}_{i}\right) ^{\varphi _{i}}\Biggr )^{{\Cap }}}, \root t^{*} \of {1-\Biggl (1-\prod \limits _{i=1}^{n}\left( 1-\mathscr {Z}_{i}\right) ^{\varphi _{i}}\Biggr )^{{\Cap }}}\Biggr )$$.

Hence, ${\Cap }$ST-r,s,t-SPFWA$\left( {\mathfrak {L}}_{1},{\mathfrak {L}}_{2},...,{\mathfrak {L}}_{n}\right) \oplus \sin {\mathfrak {L}}$

20 $$\begin{aligned} =\Biggl (\root r^{*} \of {1-\left( 1-\mathscr {X}\right) \Biggl (\prod \limits _{i=1}^{n}\left( 1-\mathscr {X}_{i}\right) ^{\varphi _{i}} \Biggr )^{{\Cap }}},\root s^{*} \of {\left( 1-\mathscr {Y}\right) \Biggl (\prod \limits _{i=1}^{n}\left( 1-\mathscr {Y}_{i}\right) ^{\varphi _{i}} \Biggr )^{{\Cap }}},\root t^{*} \of {\left( 1-\mathscr {Z}\right) \Biggl (\prod \limits _{i=1}^{n}\left( 1-\mathscr {Z}_{i}\right) ^{\varphi _{i}}\Biggr )^{{\Cap }}}\Biggr ), \end{aligned}$$

and

$$\left( ST-r,s,t-SPFWA\left( {\mathfrak {L}}_{1},{\mathfrak {L}}_{2},...,{\mathfrak {L}}_{n}\right) \right) ^{{\Cap }} \otimes \sin {\mathfrak {L}}$$

21 $$\begin{aligned} =\Biggl (\root r^{*} \of {\mathscr {X}\Biggl (1-\prod \limits _{i=1}^{n}\left( 1-\mathscr {X}_{i}\right) ^{\varphi _{i}}\Biggr )^{{\Cap }}},\root s^{*} \of {1-\Biggl (1-\prod \limits _{i=1}^{n}\left( 1-\mathscr {Y}_{i}\right) ^{\varphi _{i}}\Biggr )^{{\Cap }}\mathscr {Y}},\root t^{*} \of {1-\Biggl (1-\prod \limits _{i=1}^{n}\left( 1-\mathscr {Z}_{i}\right) ^{\varphi _{i}}\Biggr )^{{\Cap }}\mathscr {Z}}\Biggr ). \end{aligned}$$

For $\mathscr {X},\mathscr {Y},\mathscr {Z} \in [0,1]$ and a real number ${\Cap } \in [0,1]$, we have $\Biggl (\prod \limits _{i=1}^{n}\left( 1-\mathscr {Y}_{i}\right) ^{\varphi _{i}}\Biggr )^{{\Cap }},\Biggl (\prod \limits _{i=1}^{n} \left( 1-\mathscr {Z}_{i}\right) ^{\varphi _{i}}\Biggr )^{{\Cap }} \in [0,1]$. Thus by Proposition 2, we get $\mathscr {X}\Biggl (1-\prod \limits _{i=1}^{n}\left( 1-\mathscr {X}_{i}\right) ^{\varphi _{i}}\Biggr )^{{\Cap }}+\left( 1-\mathscr {Y}\right) \Biggl (\prod \limits _{i=1}^{n}\left( 1-\mathscr {Y}_{i}\right) ^{\varphi _{i}}\Biggr )^{{\Cap }}+\left( 1-\mathscr {Z}\right) \Biggl (\prod \limits _{i=1}^{n}\left( 1-\mathscr {Z}_{i}\right) ^{\varphi _{i}}\Biggr )^{{\Cap }} \le 1$

$$\implies \mathscr {X}\Biggl (1-\prod \limits _{i=1}^{n}\left( 1-\mathscr {X}_{i}\right) ^{\varphi _{i}}\Biggr )^{{\Cap }}\le 1- \left( 1-\mathscr {Y}\right) \Biggl (\prod \limits _{i=1}^{n}\left( 1-\mathscr {Y}_{i}\right) ^{\varphi _{i}}\Biggr )^{{\Cap }} \le 1- \left( 1-\mathscr {Z}\right) \Biggl (\prod \limits _{i=1}^{n}\left( 1-\mathscr {Z}_{i}\right) ^{\varphi _{i}}\Biggr )^{{\Cap }}$$

$$\implies \root r^{*} \of {\mathscr {X}\Biggl (1-\prod \limits _{i=1}^{n}\left( 1-\mathscr {X}_{i}\right) ^{\varphi _{i}}\Biggr )^{{\Cap }}} \le \root s^{*} \of {1-\left( 1-\mathscr {Y}\right) \Biggl (\prod \limits _{i=1}^{n}\left( 1-\mathscr {Y}_{i}\right) ^{\varphi _{i}}\Biggr )^{{\Cap }}} \le \root t^{*} \of {1-\left( 1-\mathscr {Z}\right) \Biggl (\prod \limits _{i=1}^{n}\left( 1-\mathscr {Z}_{i}\right) ^{\varphi _{i}}\Biggr )^{{\Cap }}}$$

Similarly, for $\mathscr {Z}, \Biggl (\prod \limits _{i=1}^{n}\left( 1-\mathscr {Z}_{i}\right) ^{\varphi _{i}}\Biggr )^{{\Cap }}.\Biggl (1-\prod \limits _{i=1}^{n} \left( 1-\mathscr {Z}_{i}\right) ^{\varphi _{i}}\Biggr )^{{\Cap }} \in [0,1]$ and by Proposition 2, we get

$$\root t^{*} \of {\left( 1-\mathscr {Z}\right) .\Biggl (\prod \limits _{i=1}^{n}\left( 1-\mathscr {Z}_{i}\right) ^{\varphi _{i}}\Biggr )^{{\Cap }}} \le \root t^{*} \of {1-\Biggl (1-\prod \limits _{i=1}^{n}\left( 1-\mathscr {Z}_{i}\right) ^{\varphi _{i}}\Biggr )^{{\Cap }}.\mathscr {Z}}$$

With the help of Eqs. (20), (21) and Definition 3, we get the result (1) which is valid for real ${\Cap } \in [0,1]$. $\square$

## Proposed decision-making Algorithm

In this section, an algorithm for the MAGDM problem is framed based on the formulated ST operators.

Suppose that ${\mathfrak {I}}=\left( {\mathfrak {I}}_{1}, {\mathfrak {I}}_{2},..., {\mathfrak {I}}_{m}\right)$ be the set of alternatives and ${\mathcal {C}}=\left( {\mathcal {C}}_{1},{\mathcal {C}}_{2},...,{\mathcal {C}}_{n}\right)$ be the set of attributes with corresponding weight vector $\varphi = (\varphi _{1},\varphi _{2},...,\varphi _{n})^{T}$ such that $\varphi _{j} >0$ and $\sum \limits _{j=1}^{n}\varphi _{j}=1$. A group of DnMs having the weight vector $\varpi = (\varpi _{1},\varpi _{2},...,\varpi _{d})^{T}$ such that $\varpi _{h}>0$ and $\sum \limits _{h=1}^{d}\varpi _{h}=1$ construct decision matrices ${\mathcal {D}}^{(h)}=\left( {\mathfrak {I}}^{(h)}_{ij}\right) _{m \times n};\; h=1,2,...,d$ using r,s,t-SPFNs.

The MAGDM problem-solving process involves the following steps.

Step 1: Collect the preferences given by DnMs in the form of decision matrix ${\mathcal {D}}=\left( {\mathfrak {I}}_{ij}^{(h)}\right)$ including r,s,t-SPFS details.
Step 2: Build the normalized decision matrix ${\mathcal {R}}= \left( p^{(h)}_{ij}\right)$ based on ${\mathcal {D}}=\left( {\mathfrak {I}}^{(h)}_{ij}\right)$, with $p_{ij}$ computed as:
  22 $$\begin{aligned} p^{(h)}_{ij}={\left\{ \begin{array}{ll} \left( \rho _{ij},\chi _{ij},\varepsilon _{ij}\right) ; \text {if benefit-type attributes} \\ \left( \varepsilon _{ij},\chi _{ij},\rho _{ij}\right) ; \text {if cost-type attributes} \end{array}\right. } \end{aligned}$$
Step 3: Integrates DnMs values ${\mathfrak {I}}_{ij}^{(h)}, h=1,2,...,d$ into ${\mathfrak {I}}_{ij}=\left( \rho _{ij},\chi _{ij},\varepsilon _{ij}\right)$ using $ST-r,s,t-SPFWA$ operator.
Step 4: Aggregate the overall rating values ${D} =\left( {{\mathfrak {I}}_{ij}}\right)$ of the alternative ${\mathfrak {I}}_{i}\left( i=1,2,...,m\right)$ into the overall assessment value ${\mathfrak {I}}_{i}=\left( \rho _{i},\chi _{i},\varepsilon _{i}\right)$ based on geometric r,s,t-SPF AO as defined in Definition 11.
Step 5: Utilizing Eq. (23) yields the score values for ${\mathfrak {I}}_{i}=\left( \rho _{i},\chi _{i},\varepsilon _{i}\right)$ where $i=1,2,...,n.$
  23 $$\begin{aligned} \mathscr {S}({\mathfrak {I}}_{i})=\frac{1}{2}\left( 1+\left( \rho _{i}^{r}-\chi _{i}^s-\varepsilon _{i}^{t}\right) \right) . \end{aligned}$$
Step 6: Arrange all alternatives ${\mathfrak {I}}_{i} \left( i=1,2,...,m\right)$ following Definition 7 and identify the most suitable alternative.

## Numerical analysis

This section offers a case study on assessing laptops to illustrate the utilization of the framework presented. It comprises four subsections: result analysis, sensitivity analysis, comparative study, and managerial implications.

### Experimental results

**Example:**

A team of three DnMs specializing in laptops convened to conduct a comprehensive decision analysis involving multiple attributes aimed at selecting the most suitable laptop model. Each criterion represents a key aspect of the laptop’s performance, while each attribute provides a finer-grained assessment of those attributes. In this context, the team has chosen the following four attributes:

Battery Life ($${\mathcal {C}}_{1}$$):
This criterion evaluates the duration for which the laptop can operate on a single battery charge. Longer battery life is generally preferred as it enhances portability and convenience.
Size ($${\mathcal {C}}_{2}$$):
Size refers to the physical dimensions of the laptop, including factors such as thickness, weight, and overall form factor. Smaller and lighter laptops are often favored for their ease of transportation.
Screen Quality ($${\mathcal {C}}_{3}$$):
The screen criterion assesses the visual display capabilities of the laptop, including factors such as resolution, color accuracy, and brightness. A high-quality screen enhances the user experience, particularly for tasks involving multimedia content.
Sound Quality ($${\mathcal {C}}_{4}$$):
Sound quality pertains to the audio output capabilities of the laptop, including speaker performance and audio clarity. Good sound quality is essential for activities such as video conferencing, multimedia playback, and general entertainment.

For this decision problem, the DnMs have the weight vector $\varpi =(0.3,0.4,0.3)^T$. Additionally, the weight vector assigned by the DnMs to the attributes is denoted as $\varphi =(0.4,0.2,0.1,0.3)^T$, indicating the relative importance of each criterion in the DM process. It’s noteworthy that all attributes are considered benefit-type attributes.

Subsequently, the team applied the proposed $r,s,t-SPF$ framework to identify the optimal model among the available options. This framework facilitates a systematic approach to MAGDM, enabling the team to effectively evaluate and compare the laptop models based on their performance across the established attributes. Through this detailed analysis, the team aims to make an informed decision that aligns with their preferences and requirements, ultimately selecting the laptop model that best fulfills their needs.

The calculation procedures are outlined as follows:

**Step 1:** The $r,s,t-SPF$ data provided by the three DnMs is presented in Tables 1, 2 and 3, respectively.

**Step 2:** Since all four attributes are benefit types. Therefore, we do not need normalization.

**Table 1:** r,s,t-SPF decision matrix ${\mathcal {D}}^{1}$.

	${\mathcal {C}}_{1}$	${\mathcal {C}}_{2}$	${\mathcal {C}}_{3}$	${\mathcal {C}}_{4}$
${\mathfrak {I}}_{1}$	$\left( 0.4,0.4,0.3\right)$	$\left( 0.5,0.7,0.2\right)$	$\left( 0.8,0.4,0.5\right)$	$\left( 0.6,0.3,0.3\right)$
${\mathfrak {I}}_{2}$	$\left( 0.5,0.6,0.3\right)$	$\left( 0.7,0.5,0.4\right)$	$\left( 0.5,0.7,0.6\right)$	$\left( 0.4,0.4,0.5\right)$
${\mathfrak {I}}_{3}$	$\left( 0.4,0.3,0.2\right)$	$\left( 0.4,0.7,0.3\right)$	$\left( 0.5,0.4,0.3\right)$	$\left( 0.8,0.4,0.8\right)$
${\mathfrak {I}}_{4}$	$\left( 0.6,0.8,0.5\right)$	$\left( 0.4,0.2,0.2\right)$	$\left( 0.5,0.7,0.8\right)$	$\left( 0.6,0.4,0.2\right)$

**Table 2:** r,s,t-SPF decision matrix ${\mathcal {D}}^{2}$.

	${\mathcal {C}}_{1}$	${\mathcal {C}}_{2}$	${\mathcal {C}}_{3}$	${\mathcal {C}}_{4}$
${\mathfrak {I}}_{1}$	$\left( 0.5,0.6,0.5\right)$	$\left( 0.2,0.4,0.3\right)$	$\left( 0.8,0.6,0.5\right)$	$\left( 0.7,0.3,0.4\right)$
${\mathfrak {I}}_{2}$	$\left( 0.8,0.3,0.5\right)$	$\left( 0.7,0.5,0.8\right)$	$\left( 0.5,0.5,0.6\right)$	$\left( 0.4,0.5,0.3\right)$
${\mathfrak {I}}_{3}$	$\left( 0.2,0.2,0.3\right)$	$\left( 0.5,0.3,0.4\right)$	$\left( 0.6,0.6,0.6\right)$	$\left( 0.6,0.7,0.4\right)$
${\mathfrak {I}}_{4}$	$\left( 0.6,0.4,0.4\right)$	$\left( 0.3,0.3,0.3\right)$	$\left( 0.6,0.7,0.3\right)$	$\left( 0.8,0.4,0.5\right)$

**Table 3:** r,s,t-SPF decision matrix ${\mathcal {D}}^{3}$.

	${\mathcal {C}}_{1}$	${\mathcal {C}}_{2}$	${\mathcal {C}}_{3}$	${\mathcal {C}}_{4}$
${\mathfrak {I}}_{1}$	$\left( 0.4,0.5,0.7\right)$	$\left( 0.8,0.5,0.7\right)$	$\left( 0.4,0.8,0.2\right)$	$\left( 0.4,0.5,0.5\right)$
${\mathfrak {I}}_{2}$	$\left( 0.7,0.6,0.5\right)$	$\left( 0.6,0.5,0.7\right)$	$\left( 0.6,0.3,0.3\right)$	$\left( 0.3,0.2,0.2\right)$
${\mathfrak {I}}_{3}$	$\left( 0.5,0.8,0.4\right)$	$\left( 0.5,0.7,0.4\right)$	$\left( 0.8,0.7,0.7\right)$	$\left( 0.7,0.3,0.6\right)$
${\mathfrak {I}}_{4}$	$\left( 0.6,0.3,0.8\right)$	$\left( 0.7,0.4,0.5\right)$	$\left( 0.8,0.4,0.4\right)$	$\left( 0.3,0.6,0.5\right)$

**Step 3:** Utilizing the experts’ weights, i.e., $\varpi =(0.3,0.4,0.3)^T$ and applying the ST-r,s,t-SPFWA operator, the collective data for each alternative is obtained and is shown in Table 4.

**Table 4:** Aggregated values of experts by ST-r,s,t-SPFWA operator.

	${\mathcal {C}}_{1}$	${\mathcal {C}}_{2}$	${\mathcal {C}}_{3}$	${\mathcal {C}}_{4}$
${\mathfrak {I}}_{1}$	$\left( 0.6460,0.1914,0.1741\right)$	$\left( 0.8201,0.1945,0.08963\right)$	$\left( 0.9175,0.2594,0.1095\right)$	$\left( 0.8208,0.09158,0.1169\right)$
${\mathfrak {I}}_{2}$	$\left( 0.9001,0.1565,0.1406\right)$	$\left( 0.8724,0.1885,0.3140\right)$	$\left( 0.7455,0.1711,0.1829\right)$	$\left( 0.5581,0.09472,0.07210\right)$
${\mathfrak {I}}_{3}$	$\left( 0.5889,0.08979,0.06281\right)$	$\left( 0.6795,0.1909,0.1017\right)$	$\left( 0.8653,0.2368,0.2029\right)$	$\left( 0.8961,0.1603,0.2446\right)$
${\mathfrak {I}}_{4}$	$\left( 0.8091,0.1564,0.2185\right)$	$\left( 0.7392,0.06270,0.07210\right)$	$\left( 0.8653,0.2703,0.1252\right)$	$\left( 0.8721,0.1537,0.1095\right)$

**Step 4:** Using the attributes’ weight vector i.e., $\varphi =(0.4,0.2,0.1,0.3)^T$ and applying the ST-r,s,t-SPFWG operator, the combined values for each alternative are obtained as follows:

$${\mathfrak {I}}_{1}=\left( 0.9174, 0.03162, 0.01721 \right)$$, $${\mathfrak {I}}_{2}=\left( 0.9083,0.01778,0.04369\right)$$,$${\mathfrak {I}}_{3}=\left( 0.8846,0.02515, 0.03093\right)$$, $${\mathfrak {I}}_{4}= \left( 0.9571, 0.03080, 0.02640\right)$$.

**Step 5:** Following Eq. (23), the score values of aggregated values of the alternatives are obtained as follows: $\mathscr {S}({\mathfrak {I}}_{1})=0.8542$, $\mathscr {S}({\mathfrak {I}}_{2})=0.8403$, $\mathscr {S}({\mathfrak {I}}_{3})=0.8062$, $\mathscr {S}({\mathfrak {I}}_{4})=0.9196$.

**Step 6:** Based on the above-derived score values, the ranking of alternatives is ${\mathfrak {I}}_{4}>{\mathfrak {I}}_{1}>{\mathfrak {I}}_{2}>{\mathfrak {I}}_{3}$.

### Influence of parameters

This section focuses on conducting a sensitivity analysis to assess how different parameters affect the ranking outcomes.

To showcase the reliability as well as uniformity of the illustration above, we check the sensitivity concerning different parameters such as $r^{*}, s^{*}$, and $t^{*}$ within a structured framework. For this, we set the values of $s^{*}=4$ and $t^{*}=3$ and explore different values for $r^{*}$. By changing the value of $r^{*}$, the ranking results of different choices stay consistent, i.e., ${\mathfrak {I}}_{4}>{\mathfrak {I}}_{1}>{\mathfrak {I}}_{2}>{\mathfrak {I}}_{3}$ which shown in Table 5. Furthermore, upon fixing $r^{*}=4, t^{*}=3$ and increase the value of $s^{*}=5,7,10,13,17,20$ in the proposed ST-r,s,t-SPFWG operator, it is noticeable that from Table 6 the ranking outcomes of choices remains unchanged i.e., ${\mathfrak {I}}_{4}>{\mathfrak {I}}_{1}>{\mathfrak {I}}_{2}>{\mathfrak {I}}_{3}$. Similarly, if we fix $r^{*}=4, s^{*}=4$ and vary the value of $t^{*}=4,7,10,13,17,20$ in ST-r,s,t-SPFWG, again, it is noticeable, that from Table 7 analogous $s^{*}$ the ranking outcomes remains same. Therefore, the proposed approach exhibits isotonicity and stability under the ST-r,s,t-SPFWG operator across various values of $r^{*},s^{*}$ and $t^{*}$.

**Table 5:** Results with different value of $r^{*}$.

$r^{*},s^{*},t^{*}$	Ranking
$r^{*}=5,s^{*}=4,t^{*}=3$	${\mathfrak {I}}_{4}>{\mathfrak {I}}_{1}>{\mathfrak {I}}_{2}>{\mathfrak {I}}_{3}$
$r^{*}=7,s^{*}=4, t^{*}=3$	${\mathfrak {I}}_{4}>{\mathfrak {I}}_{1}>{\mathfrak {I}}_{2}>{\mathfrak {I}}_{3}$
$r^{*}=10,s^{*}=4,t^{*}=3$	${\mathfrak {I}}_{4}>{\mathfrak {I}}_{1}>{\mathfrak {I}}_{2}>{\mathfrak {I}}_{3}$
$r^{*}=13,s^{*}=4,t^{*}=3$	${\mathfrak {I}}_{4}>{\mathfrak {I}}_{1}>{\mathfrak {I}}_{2}>{\mathfrak {I}}_{3}$
$r^{*}=17,s^{*}=4,t^{*}=3$	${\mathfrak {I}}_{4}>{\mathfrak {I}}_{1}>{\mathfrak {I}}_{2}>{\mathfrak {I}}_{3}$
$r^{*}=20,s^{*}=4,t^{*}=3$	${\mathfrak {I}}_{4}>{\mathfrak {I}}_{1}>{\mathfrak {I}}_{2}>{\mathfrak {I}}_{3}$

**Table 6:** Results with different value of $s^{*}$.

$r^{*},s^{*},t^{*}$	Ranking
$r^{*}=4,s^{*}=5,t^{*}=3$	${\mathfrak {I}}_{4}>{\mathfrak {I}}_{1}>{\mathfrak {I}}_{2}>{\mathfrak {I}}_{3}$
$r^{*}=4,s^{*}=7, t^{*}=3$	${\mathfrak {I}}_{4}>{\mathfrak {I}}_{1}>{\mathfrak {I}}_{2}>{\mathfrak {I}}_{3}$
$r^{*}=4,s^{*}=10,t^{*}=3$	${\mathfrak {I}}_{4}>{\mathfrak {I}}_{1}>{\mathfrak {I}}_{2}>{\mathfrak {I}}_{3}$
$r^{*}=4,s^{*}=13,t^{*}=3$	${\mathfrak {I}}_{4}>{\mathfrak {I}}_{1}>{\mathfrak {I}}_{2}>{\mathfrak {I}}_{3}$
$r^{*}=4,s^{*}=17,t^{*}=3$	${\mathfrak {I}}_{4}>{\mathfrak {I}}_{1}>{\mathfrak {I}}_{2}>{\mathfrak {I}}_{3}$
$r^{*}=4,s^{*}=20,t^{*}=3$	${\mathfrak {I}}_{4}>{\mathfrak {I}}_{1}>{\mathfrak {I}}_{2}>{\mathfrak {I}}_{3}$

**Table 7:** Results with different value of $t^{*}$.

$r^{*},s^{*},t^{*}$	Ranking
$r^{*}=4,s^{*}=4,t^{*}=4$	${\mathfrak {I}}_{4}>{\mathfrak {I}}_{1}>{\mathfrak {I}}_{2}>{\mathfrak {I}}_{3}$
$r^{*}=4,s^{*}=4, t^{*}=7$	${\mathfrak {I}}_{4}>{\mathfrak {I}}_{1}>{\mathfrak {I}}_{2}>{\mathfrak {I}}_{3}$
$r^{*}=4,s^{*}=4,t^{*}=10$	${\mathfrak {I}}_{4}>{\mathfrak {I}}_{1}>{\mathfrak {I}}_{2}>{\mathfrak {I}}_{3}$
$r^{*}=4,s^{*}=4,t^{*}=13$	${\mathfrak {I}}_{4}>{\mathfrak {I}}_{1}>{\mathfrak {I}}_{2}>{\mathfrak {I}}_{3}$
$r^{*}=4,s^{*}=4,t^{*}=17$	${\mathfrak {I}}_{4}>{\mathfrak {I}}_{1}>{\mathfrak {I}}_{2}>{\mathfrak {I}}_{3}$
$r^{*}=4,s^{*}=4,t^{*}=20$	${\mathfrak {I}}_{4}>{\mathfrak {I}}_{1}>{\mathfrak {I}}_{2}>{\mathfrak {I}}_{3}$

### Comparative study

Within this part, we provide a concise discussion comparing the developed method with well-known related techniques, such as ST-Pythagorean fuzzy weighted averaging (ST-PyFWA), ST-Pythagorean fuzzy weighted geometric (ST-PyFWG)^28^, ST-q-rung orthopair fuzzy weighted averaging (ST-q-ROFWA), ST-q-rung orthopair fuzzy weighted geometric (ST-q-ROFWG)^30^, ST-p,q-quasirung orthopair fuzzy weighted averaging (ST-p,q-QOFWA), ST-p,q-quasirung orthopair fuzzy weighted geometric (ST-p,q-QOFWG)^35^, ST-spherical fuzzy weighted averaging (ST-SPFWA),ST-spherical fuzzy weighted geometric (ST-SPFWG)^28^, ST-t-spherical fuzzy weighted averaging (ST-TSPFWA), ST-t-spherical fuzzy weighted geometric (ST-TSPFWG)^36^, r,s,t-spherical fuzzy weighted averaging (r,s,t-SPFWA), r,s,t-spherical fuzzy weighted geometric (r,s,t-SPFWA)^18^, r,s,t-SPF Aczel-Alsina weighted averaging (r,s,t-SPFAAWA), r,s,t-SPF Aczel-Alsina weighted geometric (r,s,t-SPFAAWG)^19^. The results acquired are displayed in Table 8.

**Table 8:** Ranking order of the alternatives using different approaches.

Methods	Ranking values
Proposed approach	${\mathfrak {I}}_{4}>{\mathfrak {I}}_{1}>{\mathfrak {I}}_{2}>{\mathfrak {I}}_{3}$
ST-q-ROFWA^30^	${\mathfrak {I}}_{2}>{\mathfrak {I}}_{4}>{\mathfrak {I}}_{1}>{\mathfrak {I}}_{3}$
ST-q-ROFWG^30^	${\mathfrak {I}}_{4}>{\mathfrak {I}}_{1}>{\mathfrak {I}}_{2}>{\mathfrak {I}}_{3}$
ST-p,q-QOFWA^35^	${\mathfrak {I}}_{2}>{\mathfrak {I}}_{4}>{\mathfrak {I}}_{1}>{\mathfrak {I}}_{3}$
ST-p,q-QOFWG^35^	${\mathfrak {I}}_{4}>{\mathfrak {I}}_{1}>{\mathfrak {I}}_{2}>{\mathfrak {I}}_{3}$
ST-TSPFWA^36^	${\mathfrak {I}}_{2}>{\mathfrak {I}}_{4}>{\mathfrak {I}}_{1}>{\mathfrak {I}}_{3}$
ST-TSPFWG^36^	${\mathfrak {I}}_{4}>{\mathfrak {I}}_{1}>{\mathfrak {I}}_{2}>{\mathfrak {I}}_{3}$
r,s,t-SPFWA^18^	${\mathfrak {I}}_{2}>{\mathfrak {I}}_{4}>{\mathfrak {I}}_{3}>{\mathfrak {I}}_{1}$
r,s,t-SPFWG^18^	${\mathfrak {I}}_{4}>{\mathfrak {I}}_{1}>{\mathfrak {I}}_{2}>{\mathfrak {I}}_{3}$
r,s,t-SPFAAWA^19^	${\mathfrak {I}}_{2}>{\mathfrak {I}}_{4}>{\mathfrak {I}}_{3}>{\mathfrak {I}}_{1}$
r,s,t-SPFAAWG^19^	${\mathfrak {I}}_{4}>{\mathfrak {I}}_{1}>{\mathfrak {I}}_{3}>{\mathfrak {I}}_{2}$
ST-PFWA^28^	Unable to aggregate
ST-PFWG^28^	Unable to aggregate
ST-SPFWA^29^	Unable to aggregate
ST-SPFWG^29^	Unable to aggregate

Based on the data presented in this Table 8, it can be deduced that the optimal alternative identified by the proposed method aligns with the majority of existing approaches, thus affirming the validity of the proposed approach. We can further notice that ST-AOs of PyFS and SPFS cannot handle the data provided in the current problem. The analysis suggests that the existing AOs can be viewed as specific instances within the framework of the proposed method. Furthermore, this outcome indicates that the proposed method offers a broader approach than the existing AOs.

Based on the comparative analysis, the merits and outcomes of the reported framework are outlined as follows:

(i). Compared to other assessment frameworks, the framed approach utilizes more reasonable input data, namely r, s, t-SPFNs, for evaluating alternatives. However, the data in the other assessment frameworks^28–30,35,36^ doesn’t take advantage of the three flexible parameters r, s, and t. The inclusion of these adjustable parameters broadens the scope of the application and allows for a more reasonable capture of data.
(ii). Unlike the existing methods^18,19^, our proposed ST operators take into account the significance of trigonometry’s characteristics, such as its periodicity and symmetry, in the analysis. This makes our approach superior to the existing r,s,t-SPF AOs.
(iii). The framework presented requires only a few straightforward steps, highlighting its computational convenience. This accessibility makes it highly suitable for emergency decision support scenarios, where quick and effective DM is crucial.

The proposed study also exhibits certain drawbacks, which are enumerated as follows:

I. One limitation of the outlined framework is that it requires prior knowledge of the weights for DnMs and attributes. Without this information upfront, the algorithm isn’t applicable, which could be problematic for scenarios where these details aren’t available beforehand.
II. The developed ST AOs lack the capability to account for divisions among input arguments and may not be deemed valid in MAGDM problems, where attributes can be classified into distinct classes.

### Managerial implications

The above analysis of laptop selection provides a comprehensive overview of the most commonly used technologies in the industry, highlighting their respective advantages and drawbacks. The proposed MAGDM framework effectively identifies the most pertinent laptop options, emphasizing those that are environmentally friendly, cost-effective, user-friendly, and capable of meeting substantial computing needs. This information offers valuable insights for both businesses and consumers, enabling them to make informed decisions based on their specific requirements. Additionally, policymakers can leverage this research to promote the adoption of laptops as essential tools for education, business, and personal use, particularly in underserved areas where access to traditional power sources may be limited. The widespread adoption of laptops and the advancement of their technological recycling processes hold the potential to stimulate industrial growth and create employment opportunities in the technology sector.

### Ethical approval

This material is the authors’ own original work, which has not been previously published elsewhere.

## Conclusion

The primary objective of this study was to introduce a novel perspective on operational laws and operators applicable to various *r*, *s*, *t*-SPFNs. We introduced STOLs and defined a new ST-*r*, *s*, *t*-SPFN to address this aim. Detailed discussions were conducted on the fundamental properties of these proposed laws. Additionally, we formulated several weighted averaging and geometric operators based on these laws to aggregate *r*, *s*, *t*-SPF information. The relationships between these operators were analyzed through derived inequalities, elucidating their correlations. The basic axioms of these operators were demonstrated to be satisfied within the proposed framework. Moreover, to tackle group DM problems, we developed a novel MAGDM algorithm, which considers multiple decision-makers and alternatives within a *r*, *s*, *t*-SPF environment. The reliability and effectiveness of the developed algorithm were evaluated through a numerical example and compared with existing approaches. Through these analyses, it was observed that the presented algorithm and operators effectively manage a broader spectrum of information, rendering them highly capable of addressing DM Problems.

In the future, we aim to address the limitations of this study, as highlighted in the analysis section. To this end, we intend to integrate additional operators, such as the Maclaurin symmetric mean operator, the partition aggregation operator, and the power Muirhead mean operator, with the proposed ST AOs. Subsequently, we plan to develop an integrated weight calculation method by combining some subjective weighting methods, such as the level-based weight assessment method or the rank sum method, with an entropy-based approach.

## Data Availability

All data generated or analysed during this study are included in this published article.
